# Gap Junction Enhancer Increases Efficacy of Cisplatin to Attenuate Mammary Tumor Growth

**DOI:** 10.1371/journal.pone.0044963

**Published:** 2012-09-13

**Authors:** Stephanie N. Shishido, Thu A. Nguyen

**Affiliations:** Departments of Diagnostic Medicine/Pathobiology, Kansas State University, Manhattan, Kansas, United States of America; University of Medicine and Dentistry of New Jersey, United States of America

## Abstract

Cisplatin treatment has an overall 19% response rate in animal models with malignant tumors. Increasing gap junction activity in tumor cells provides the targets to enhance antineoplastic therapies. Previously, a new class of substituted quinolines (PQs) acts as gap junction enhancer, ability to increase the gap junctional intercellular communication, in breast cancer cells. We examined the effect of combinational treatment of PQs and antineoplastic drugs in an animal model, showing an increase in efficacy of antineoplastic drugs via the enhancement of gap junctions. Mice were implanted with estradiol-17ß (1.7 mg/pellet) before the injection of 1×10^7^ T47D breast cancer cells subcutaneously into the inguinal region of mammary fat pad. Animals were treated intraperitoneally with DMSO (control), cisplatin (3.5 mg/kg), PQ (25 mg/kg), or a combining treatment of cisplatin and PQ. Cisplatin alone decreased mammary tumor growth by 85% while combinational treatment of cisplatin and PQ1 or PQ7 showed an additional reduction of 77% and 22% of tumor growth after 7 treatments at every 2 days, respectively. Histological results showed a significant increase of gap junction proteins, Cx43 and Cx26, in PQ-treated tissues compared to control or cisplatin. Furthermore, evidence of highly stained caspase 3 in tumors of combinational treatment (PQ and cisplatin) was seen compared to cisplatin alone. We have showed for the first time an increase in the efficacy of antineoplastic drugs through a combinational treatment with PQs, a specific class of gap junction enhancers.

## Introduction

Breast cancer is the most common cancer in women worldwide and mortality from breast cancer is consistent due to tumor metastasis [1]. Defects in neoplastic cells, such as excess proliferation, invasion, and metastasis, have a crucial role in the loss of tissue homeostasis [2]–[4]. Gap junctions are the only communicating junctions found in animal tissues, in all species, which are responsible for the direct trafficking of ions and molecules with molecular weights less than 1,200 Daltons [5]. Gap junctions directly connect the cytoplasms of neighboring cells, to allow the passage of intercellular signaling molecules and homeostatic regulators such as anti-growth signals and apoptotic factors. Intercellular junctions are important in the maintenance of the cellular homeostasis, cell differentiation, and cellular death. A main characteristic of cancer formation is the loss of gap junction intercellular communication (GJIC) through the decreased expression or absence of gap junctions [6].

Restoring GJIC in tumor cells is one approach that increases the spread of cytotoxic drugs and subsequently enhances antineoplastic therapies. Use of a gap junction enhancer may potentiate the bystander effect of cytotoxic compounds, such as cisplatin and paclitaxel. Recently, a new class of substituted quinolines (PQs) was synthesized and found to possess potent inhibitory activities against T47D breast cancer cells (IC_50_ value of PQ7 is 16 nM and PQ1 is 119 nM) through the enhancement of GJIC [7]–[8]. PQ7 has the ability to enhance the GJIC between neoplastic cells by increasing the expression of connexin 43 (Cx43) [9]. In addition, *in vivo*, the treatment of PQ7 on nude mice with T47D xenografts showed a 100% decrease in tumor growth after seven intraperitoneal injections [9]. This agent is capable of normalizing GJIC and has cancer-preventive properties.

Cisplatin is one of the most widely used cancer chemotherapeutic agents used clinically, but renal failure is a common problem in patients. Cisplatin nephrotoxicity is dose-related and used to be considered dose limiting [10]. The primary mechanism for cisplatin toxicity is via formation of platinum-DNA adducts that induce cell cycle arrest [11], [12]. Other main mechanisms of action include DNA-protein cross linking, ROS generation leading to oxidative stress [13], and a gap junction-mediated cell-interdependent pathway [14]. The cell interdependent pathway of cisplatin toxicity requires DNA dependent protein kinase (PK) signaling and intercellular communication through gap junctions [14]. He et al. [15] showed that Cx32-composed gap junctions are required components of toxicity suggesting a dependence on cells being GJIC competent. Cisplatin damage in one cell triggers DNA-PK dependent signal and is transmitted by GJIC to neighboring cells. Jensen and Glazer [14] showed that by inhibiting GJIC with lindane, immortalized mouse embryonic fibroblasts (MEFs) were protected from cisplatin toxicity, while increasing GJIC by transfecting MCF-7 breast cancer cells with Cx43 enhanced drug sensitivity. Induction of apoptosis/necrosis from cisplatin in one cell may cause a “death signal” that is transmitted to neighboring cells through gap junctions.

Increasing gap junction activity or enhancing GJIC in tumor cells provides the targets to enhance antineoplastic therapies. Tanaka and Grossman [16] showed that by transfecting human bladder cancer cells with Cx26, tumor formation could be prevented. In combination with cisplatin an increase in GJIC promoted apoptosis, cell cycle arrest, and down regulated BCL-2 [16]. A new class of substituted quinolines (PQs) possesses inhibitory activities against breast cancer cells through the enhancement of GJIC. The objective of this study was to examine the effect of combinational treatment of PQ and antineoplastic drugs in a xenograft tumor model, showing an increase in efficacy of the antineoplastic drug, cisplatin (cis-diamminedichloroplatinum), via the enhancement of gap junctions.

## Materials and Methods

### Ethics Statement

Husbandry of animals is conducted by the Comparative Medical Group (CMG) at the College of Veterinary Medicine at Kansas State University. The CMG animal facilities are fully accredited by the Association for Assessment and Accreditation of Laboratory Animal Care, International (AAALAC). The compliance to aspects of animal welfare law is regularly monitored by the veterinary staff. Animal care and use protocols were approved by the Institutional Animal Care and Use Committee (IACUC) at Kansas State University (Protocol Number: 2985), Manhattan following NIH guidelines.

### Compounds

Compounds PQ1, 6-Methoxy-8-[(3-aminopropyl)amino]-4-methyl-5-(3-trifluoromethyl-phenyloxy)quinolines, and PQ7, 6-methoxy-8-[(2-furanylmethyl)amino]-4-methyl-5-(3-trifluoromethylphenyloxy)quinoline, were graciously provided by Dr. Duy H. Hua (Kansas State University, Manhattan, KS). Cisplatin, *cis*-Diamminedichloroplatinum (II), was purchased from Sigma Aldrich (St. Louis, MO).

### Cell Line and Cell Culture

The T47D human breast cancer cell line was purchased from American Type Cell Culture (ATCC, Manassas, VA). Cells were grown in RPMI medium supplemented with 10% fetal bovine serum (Atlanta Biologicals, Lawrenceville, GA) at 37°C with 5% CO_2_ in T-125 cm^2^ flasks.

### Xenograft Tumors of T47D Cells in Nude Mice

Nu/Nu mice were ordered from Charles River Laboratories International, (Wilmington, MA, USA) and implanted with 17-β-estradiol (1.7 mg/pellet, Innovative Research of America, Sarasota, FL) before injection of 1×10^7^ T47D breast cancer cells subcutaneously into the inguinal region of the mammary fat pad. Cell viability of T47D cells was performed prior to the injection. Tumor size was measured in two dimensions with calipers every 2 days starting at day 7. Mice were observed for any change in behavior, appearance or weight. When tumors reached >50 mm^3^, six animals were randomly assigned to each treatment group. Mice were administered 25 mg/kg PQ1 or PQ7 in succinic acid salt, 3.5 mg/kg cisplatin, or a combination of PQ and cisplatin via intraperitoneal injection of 100 µl. Compounds were dissolved in DMSO, which was used as a vehicle control at the same volume.

### Western Blot Analysis

Tissue was harvested from the mice and whole cell extractions conducted using lysis buffer (20 mM Tris pH 7.5, 0.5 mM EDTA, 0.5 mM EGTA, and 0.5% Triton X-100) with 1∶1000 dilution of protease inhibitors (Sigma-Aldrich, Saint Louis, MO, USA). Tissue was homogenized via the OMNI Bead Ruptor 24 at a speed of 5.65 m/s for 45 seconds, followed by centrifugation at 13,000 rpm for 30 minutes at 4°C. Twenty-five µg of whole-cell extract was resolved by 10% SDS polyacrylamide gel electrophoresis (PAGE) and transferred to nitrocellulose membrane (Midwest Scientific, Saint Louis, MO, USA). Nitrocellulose membrane was blocked in 5% milk for an hour at room temperature and then incubated with monoclonal antibodies against anti-Cx43, -Cx32, -Cx26, -caspase 3, -caspase 8, and -caspase 9 (Santa Cruz Biotechnologies, Santa Cruz, CA, USA) and actin (Sigma-Aldrich) at a dilution of 1∶1,000. Western blots were detected by enhanced chemiluminescence detection reagents (Pierce, Rockford, Illinois, USA) and visualized by Fluorochem E imaging system (ProteinSimple).

### Immunohistochemistry

All tumors were removed and fixed in a solution of 10% formaldehyde and embedded into paraffin prior to sectioning them onto slides at a 5 µm thickness. Paraffin sections (5 µm) were dried at 60°C for 25 minutes. Deparaffinization was performed with 100% xylene and 100%, 90%, 75%, 50% ethanol. Antigen retrieval was performed in 1× citrate buffer solution and steam. Slides were then incubated overnight at room temperature with a polyclonal antibody (1∶50 dilution). Antibodies include: connexin 43, 32, 26; caspase 3, 8, 9; survivin; Cyclin D1; Ki-67; and Mig (Santa Cruz Biotechnologies, Santa Cruz, CA, USA). After washes in PBS, slides were successively incubated with biotinylated secondary antibodies (1∶1000) for 15 minutes. Slides were washed and immunostains were amplified by incubation with Avidin Biotin Complex (ABC) for 10 minutes accordingly. Cells were visualized with 3,3-diaminobenzidine (DAB) followed by a hematoxylin counterstain. The sections were viewed and the images captured with a Nikon 80i microscope under 40× and 60× magnification.

### Statistical Analysis

Significance was considered at a *p*-value ≤0.05 using Student’s t-test analysis. All data are presented as mean ±95% confidence interval of at least three independent experiments.

## Results

### T47D Xenograft Tumor Growth in Nude Mice

**Figure 1:**
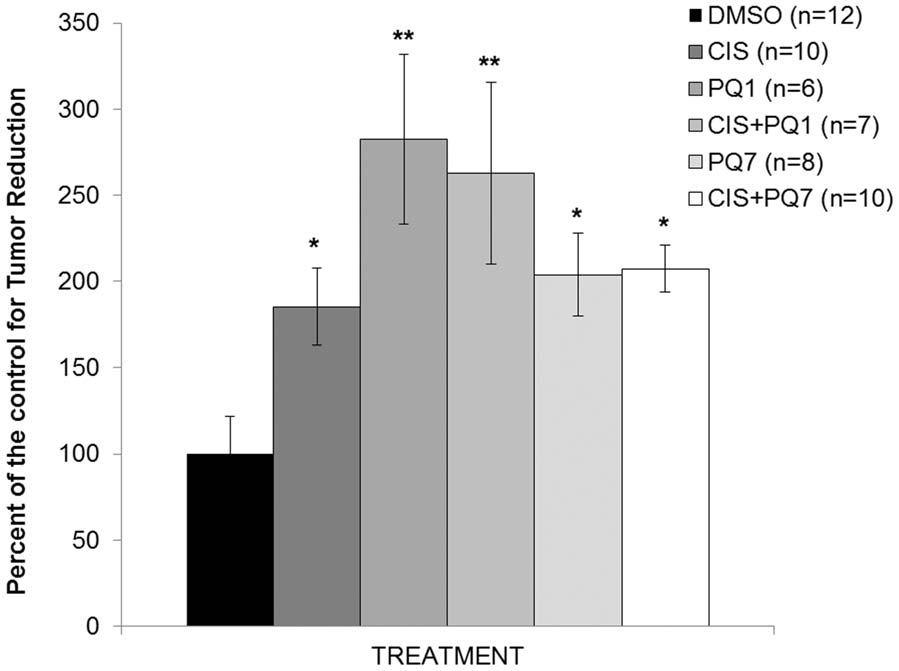
Xenograft Tumor Growth in Nude Mice. The graphical presentation shows the proportion of the percent reduction in tumor volume normalized to control after 7 IP injections of DMSO, cisplatin (3.5 mg/kg), PQ1 (25 mg/kg), PQ7 (25 mg/kg), or a combination of cisplatin and PQ. *P-value is <0.05 compared to control. **P-value is <0.05 compared to control and cisplatin treatments.

Mice were implanted with 17ß-estradiol (1.7 mg/pellet) before the injection of 1×10^7^ T47D breast cancer cells subcutaneously into the inguinal region of mammary fat pad. Seven days post cell injection, animals was randomly assigned to each treatment group. Animals were treated intraperitoneally with DMSO as a control of drug solvent, cisplatin, PQ1, PQ7, or a combining treatment of cisplatin and PQ in a total volume of 100 µl. All treatments significantly reduced tumor size (Figure 1) compared to control. Cisplatin alone decreased mammary tumor growth by 85% while combinational treatment of cisplatin and PQ1 showed an additional 77% reduction after 7 treatments at every 2 days (*p*-value of 0.012). PQ1 further decreased tumor growth after seven injections by 97% compared to cisplatin treatment alone with a *p*-value of 0.001. The data demonstrates that PQ7 alone and in combination with cisplatin significantly reduced T47D xenograft tumor group compared to control (P-values <0.001). With PQ7 alone, there was an additional 19% reduction in tumor size compared to cisplatin. Combinational treatment further decreased T47D tumor size by 77% and 22% for PQ1 (P-value: 0.028) and PQ7 combinations with cisplatin, respectively, compared to cisplatin alone. Combinational treatment of cisplatin with PQs showed greater reductions in tumor volume compared to cisplatin alone.

### Protein Expression of Xenograft Tumors

**Figure 2:**
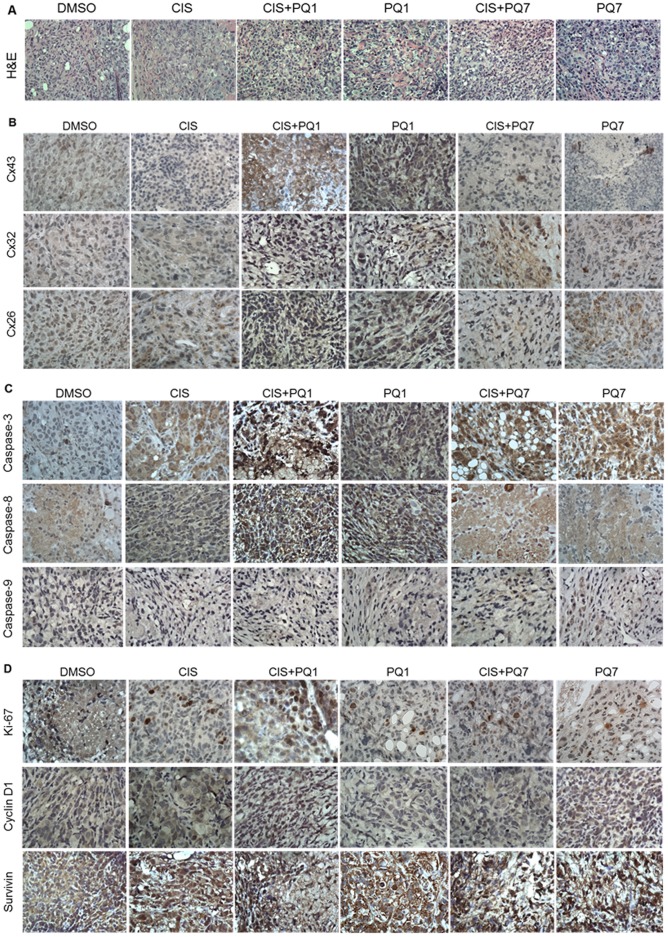
Immunohistochemistry of T47D xenograft tumors. Protein expression of A) Hemoxylin and Eosin staining under 40× magnification, B) gap junction proteins (Cx 43, 32, and 26) under 60× magnification, C) apoptotic proteins (caspase-3, -8, and -9) under 60× magnification and D) proliferative (Ki-67 and Cyclin D1) and survival proteins under 60× magnification in T47D xenograft tumors harvested after 7 IP injections treated with either DMSO (control), cisplatin, PQ1, PQ7, or a combination of cisplatin and PQs.

Morphological changes are the basis for contemporary cancer diagnosis. Hematoxylin and eosin (H&E) staining showed consistent morphology of all xenografts despite treatment received (Figure 2A). Tumor sections showed a solid nest of predominately poorly-differentiated tumor cells with large, irregular nuclei, coarse granular chromatin, prominent nucleoli, and high mitotic activity. Neoplastic cells were larger than normal epithelium with a characteristic epithelioid morphology and marked nuclear pleomorphism. Histological staining does not show any prominent features of apoptosis or necrosis for any treatment group.

**Figure 3:**
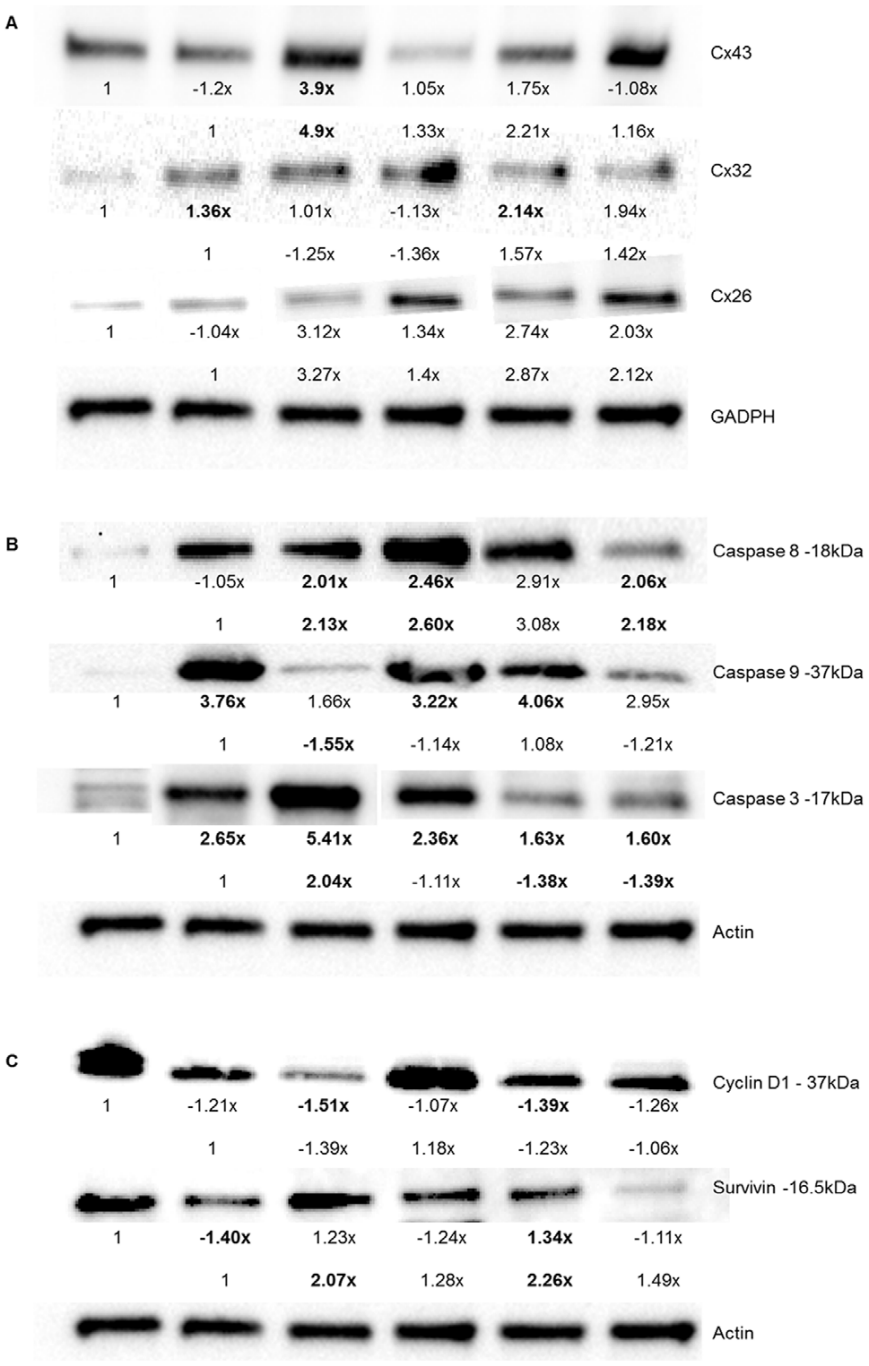
Protein expression of T47D xenograft tumors. Protein expression of A) gap junction proteins (Cxs), B) apoptotic proteins (caspases), C) Cyclin D1 and survivin from T47D xenograft tumors harvested after 7 IP injections treated with either DMSO (control), cisplatin, PQ1, PQ7 or a combination of cisplatin and PQs. Actin and GADPH are loading controls. Numbers indicate the fold difference from control (top row) and cisplatin (bottom row). x = fold induction. **Bold** P-value is <0.05 compared to control or cisplatin (n = 3).

Adjacent cells are able to exchange homeostatic regulators, such as anti-growth signals and apoptotic factors, through hydrophilic gap junction channels. Each gap junction is composed of two hemichannels (connexons) that are embedded in the plasma membrane. These connexons are formed by six connexin proteins [3], of which there are 21 different human connexin genes identified [17]. Only three connexin proteins are expressed in the human breast tissue: Cx43, Cx32, and Cx26 [18]. Immunoblot analysis and immunohistochemistry were conducted on T47D xenograft tumors harvested from mice after 7 intraperitoneal injections of DMSO, cisplatin, PQ1, PQ7, or a combining treatment of cisplatin and PQ. Tumors treated with PQ alone and in combination showed an increase in gap junction proteins (connexin 43, 32, and 26), compared to controls and cisplatin treated tumors (Figure 2B). Cisplatin treatment decreased the expression of Cx43 compared to control (Figure 2B). Western blot analysis of tumor homogenates showed that PQ1 significantly increased Cx43 expression in T47D xenografts by a 3.9 fold increase compared to control (p-value  = 0.003) and 4.9 fold change compared to cisplatin (p-value: 0.007) treated mice (Figure 3A).

The apoptotic signaling pathway induced by the treatment was determined by analysis of caspase expression. Two major signaling pathways lead to apoptosis. One is mitochondrial release of pro-apoptotic effectors such as caspase-9, which leads to caspase-dependent or independent apoptosis [19]. The other involves the interaction of death receptors with associated proteases and activation of caspase-8 [20]. Data indicate there is an increase in the density of apoptotic proteins (caspase-3, -8, and -9) staining with PQ treatment compared to control and cisplatin alone (Figure 2C), which is confirmed by Western blot analysis (Figure 3B). Cisplatin treatment increased capase-9 expression by 3.7× (P-value: 0.007) and caspase-3 expression by 2.7 fold (P-value: 0.0004) in T47D xenografts compared to control (Figure 3B). PQ1 treatment increased caspase-3,-8,-9 expression in tumors compared to control by a 5.4 fold (P-value <0.0001), 2.0 fold (P-value: 0.003), and 1.6 fold change respectively. Compared to cisplatin treatment PQ1 increased caspase-3 expression by 2.0 fold (P-value: 0.0007). Additionally PQ7 also increases caspase-3, -8, and -9 expression compared to control by 1.6 fold (P-value: 0.015), 2.8 fold, and 3.8 fold (P-value: 0.001) respectively. This suggests that PQs upregulate the expression of apoptotic signaling molecules to increase cellular induced death. Combinational treatment of PQs and cisplatin did not increase caspase-3 or -9 expressions significantly from cisplatin alone. Caspase-8 expression was significantly increased with combinational treatment of PQ and cisplatin by 2.6 fold (P-value: 0.02) and 2.2 fold (P-value: 0.01) for PQ1 and PQ7 combinations, respectively. There is a significant increase in apoptosis in PQ7 treated cells compared to those treated with cisplatin alone, but the tumor sizes between groups are not significantly different.

Proteins that inhibit apoptosis provide protection for tumor cells against cytotoxic compounds. Survivin is a member of the inhibitors of apoptosis protein family that is expressed during embryogenesis and in tumor cells as an anti-apoptotic protein that is capable of regulating mitosis [21]–[23]. Survivin is highly expressed in a range of tumors and its expression correlates with both accelerated relapse and chemotherapy resistance [24]. T47D xenograft tumors were isolated after treatment to determine the expression of survivin. All tumors that received treatment shows an intense immunohistological staining for survivin, but expression was not evenly distributed through the tissue similar to the control (Figure 2D). Western blot analysis of the tumors indicates cisplatin treatment significantly reduced survivin expression compared to control by 1.4 fold (P-value: 0.02; Figure 3C). PQ1 treatment increased survivin expression by 2.1 fold (P-value: 0.04) compared to cisplatin. PQ7 showed a 0.3× increase in survivin expression compared to control (P-value: 0.006) and a 2.25 fold increase compared to cisplatin treatment (P-value: 0.0045).

Ki-67 and Cyclin D1 were used as biomarkers for cell proliferation. Ki-67 is found in rapidly dividing cells and is used to determine the rate of cellular proliferation. Cyclin D1 is a key cell cycle regulator in which over expression results in rapid progression from G1 to S phase in mitosis [25]. From immunohistochemistry all isolated xenograft tumors that were treated showed a decreased expression in Ki-67 (Figure 2D). The treated tumors have less cells expressing Ki-67, but those cells that are expressing this protein show an upregulation compared to control. Cyclin D1 expression in T47D xenografts were significantly lower with PQ treatment compared to control by 1.5 fold (P-value 0.0007) and 0.4× (P-value 0.008) for PQ1 and PQ7 respectively (Figure 2D and 3C). This indicates that PQ treatment downregulates the expression of proliferative proteins Ki-67 and Cyclin D1 in xenograft tumors.

### Histological Study of Metabolic Organs

**Figure 4:**
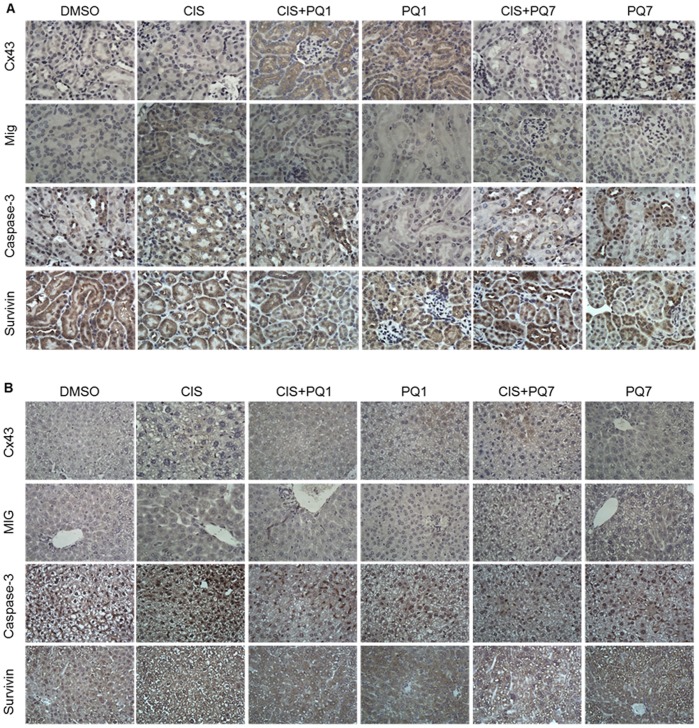
Immunohistochemistry of metabolic organs from nude mice. Protein expression of gap junction protein Cx43, the monokine induced by IFN-gamma (MIG), apoptotic protein (caspase 3) and survivin in A) kidney and B) liver from mice with T47D xenograft tumors harvested after 7 IP injections treated with either DMSO (control), cisplatin, PQ1, PQ7 or a combination of cisplatin and PQs (view image under 60× magnifcation).

Histological examination of the kidney and liver from xenografted mice showed no significant difference in morphology due to the treatment received. To determine if there was any change in protein expression of the metabolic organs, immunoblot analysis and immunohistochemistry was conducted. In the kidney there was an increase in Cx43 expression and a decrease in survivin expression due to PQ treatment (Figure 4A). There was an increase in caspase 3 expression after cisplatin treatment, which was not surprising since nephrotoxicity is common with cisplatin treatment. The kidney showed a decrease in caspase 3 expression with PQ1 treatment. The liver showed an increase in caspase-3 expression with cisplatin treatment (Figure 4B), suggesting possible cisplatin-induced hepatotoxicity. PQ treatment in combination with cisplatin appeared to decrease caspase-3 expression compared to cisplatin alone. There was an additional increase in survivin expression due to PQ treatment. There was no significant difference in Cx43 expression of the liver between treatment groups.

The monokine induced by interferon-gamma (MIG) was used as a biomarker for inflammatory signaling, indicating cytotoxicity due to treatment. The enhanced release of this CXC chemokine targets activated T cells, causing an increase in intracellular calcium ion concentrations and chemotaxis [26]. The kidney isolated from cisplatin treated animals had an increase in MIG expression (Figure 4A). The combinations of cisplatin and PQ show less staining than cisplatin alone, while PQs alone show low levels of MIG expression. The liver showed a similar pattern of staining for MIG expression with each treatment (Figure 4B). The decrease in MIG expression suggests that PQs may provide protection from the inflammatory response of cisplatin treatment.

**Figure 5:**
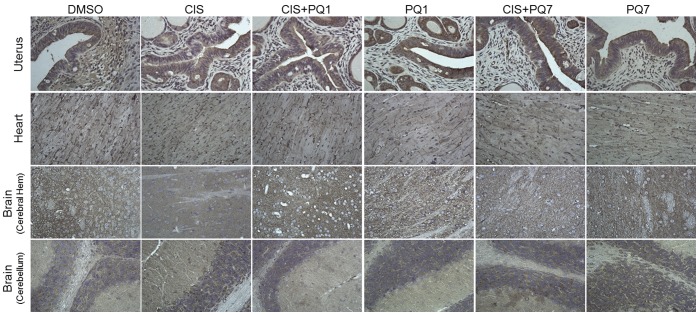
Immunohistochemistry of organs harvested from nude mice. Protein expression of gap junction protein Cx43 in the uterus, heart, and brain (cerebral hemisphere and cerebellum) from mice with T47D xenograft tumors harvested after 7 IP injections treated with either DMSO (control), cisplatin, PQ1, PQ7 or a combination of cisplatin and PQs.(view image under 40× magnification).

To determine if the normal GJIC of various organs was potentially affected by treatment, the expression of Cx43 was observed. The uterus, heart, and brain showed no change in Cx43 expression with any treatment (Figure 5). There is no observable deleterious effect due to an increase in connexin expression of cells in the liver and kidney. Further studies must be made to determine the full effects of this response.

## Discussion

This study used T47D xenografts to determine the effects of the combinational treatment of cisplatin and gap junction enhancers, PQs, in tumor-bearing mice. The results showed a decrease in tumor growth with PQ treatment, both alone and in combination with cisplatin, compared to control after seven injections (Figure 1). The combination of PQ1 and cisplatin significantly reduced tumor size compared to cisplatin alone, providing evidence that PQ1 can increase the efficacy of antineoplastic drugs in this animal model. Previously PQ7 demonstrated the ability to enhance GJIC activity through an increase in connexins 43 expression [9]. Here we show an increased expression of connexins 43, 32, and 26, suggesting a corresponding increase in cell to cell communication which would allow more efficient trafficking of cisplatin. Protein expression in the tissue sections indicates that connexin 43 is being regulated by PQ treatment. Results support previous data [27] that cisplatin cytotoxicity is dependent on GJIC. There is an increase in cisplatin-mediated response with GJIC enhancement.

The decrease in tumor size seen with PQ treatment may be attributed to an increase in apoptosis as the result of an up-regulation of caspase-3, caspase-8, and caspase-9 (Figure 2B). Western blot analysis showed a significant increase in caspase-3 expression of PQ1 treated tumors compared to control (Figure 3). These findings suggest that PQ1 and PQ7 are anticancer agents. Cisplatin treatment did not upregulate caspase-8 expression since cisplatin induced apoptosis is regulated by caspase-9 [28]. Additionally caspase expression was not downregulated with cisplatin treatment, indicating that there was not cisplatin resistance observed with the T47D xenograft tumors. There was an increase in caspase-8 expression in T47D xenograft tumors of PQ treated mice compared to cisplatin and control tumors, showing that PQ induced apoptosis through induction of both caspase-8 and capase-9 signaling.

Apoptosis is recognized as a major mode of cisplatin induced cell death. From histological results, there is a significant increase of apoptosis in PQ-treated cells compared to those treated with cisplatin alone. The tumor sizes between cisplatin alone, PQ7 alone, and cisplatin and PQ7 in combination treated groups are not significantly different despite the differences in caspase expression. The process of apoptosis produces multiple distinct populations of cells at varying stages, from early stage to secondary necrosis [29], [30]; therefore, the discrepancy in tumor size may be due to insufficient clearance from the body within the 14 day time period. Gap junctions have previously been shown to induce the synchronous cell death behavior of coupled cells [31], suggesting that PQs affect cell death by increasing connexin expression and indirectly inducing both pathways of apoptosis through an increase in caspase expression.

Western blot analysis resulted in highly variable protein expression with whole organ homogenates, most likely due to the presence of multiple tissue types in each organ. Kadle et al. [32] showed that different forms of Cx43 have a tissue-specific distribution, suggesting tissue wide differences in protein expression. More data on the tissue-specific distribution of proteins, specifically connexins, is needed to accurately determine the effects of utilizing a gap junction enhancer systemically.

Combinational treatment of cisplatin and PQ was not significantly different from PQs alone. Wang *et al.* [27] demonstrated that high cisplatin concentrations strongly inhibit GJIC through direct interactions with connexins and indirect reduction of connexin expression. Cisplatin thus may act as a competitor for gap junction enhancers. PQ7 may not have significantly increased the efficacy of cisplatin in a combinational treatment due to the direct competition between the compounds have when targeting gap junctions. Cisplatin was shown to inhibit Cx32/Cx26 heteromeric hemichannel in a concentration dependent manner [27], which is supported by the data presented in immunohistochemistry and immunoblot of Cx32 and Cx26 (decrease in expression).

Cisplatin induced “death signal” is transmitted to neighboring cells via GJIC [14]. Enhancement of GJIC may allow transmission of this “death signal” more efficiently between cells. Peterson-Roth et al. [33] showed that the level of expressed Cx43 in a cancer cell modulates cell-to-cell cisplatin-mediate response. Overexpression and activation of src is seen clinically in many cancer types treated with cisplatin [34]–[36]. Oncoproteins, such as src, promote cell growth and survival when exposed to cytotoxic agents [37]–[39], therefore protecting the cancer cells from chemotherapeutics. Src is specifically induced by cisplatin to produce tyrosine phosphorylation of Cx43, decease GJIC, and increases cell survival; thus, elevates survival of the neighboring cells by disrupting GJIC of the “death signal” [33]. Enhancement of GJIC with PQ may counter the effects of src in the cancer cell to increase the efficacy of cisplatin through an increase in transmission of the “death signal”. The fact that cancer cells are widely accepted to be deficient in GJIC and/or connexins and that cisplatin inhibits GJIC may contribute to the development of cisplatin resistant tumors. Development of drugs and methods that can increase or recover GJIC may be a new potent way to enhance chemotherapeutic methods and radiotherapy, which has also been shown to be GJIC-dependent [31].

Renal failure in cancer patients is a common problem. Cisplatin nephrotoxicity is clearly dose-related and increases with frequency of administration and cumulative dose [40]. The increased cell-to-cell communications displayed in the tumor cells is seen in the kidney and liver, but not in any other vital organ. There are no observable morphological or molecular abnormalities due to the increase in connexin expression. Cisplatin treatment alone induced an increase in both caspase and MIG expression, while PQ treatment did not (Figure 5). The combinational treatment conducted in this study shows that PQs can be utilized to decrease the cytotoxicity of cisplatin. This provides evidence for a new combinational treatment for breast cancer using cisplatin at a reduced dose to prevent renal toxicity. Present data leads to the idea that PQ, at lower concentrations than needed for anticancer effects, may improve the efficacy of chemotherapeutic agents. This would allow the use of lower drug concentrations, thus decreasing the extent of detrimental side effects due to the cytotoxicity of the compounds.

Gap junction enhancers prove to accelerate apoptotic cell death in breast cancer tumor cells while increasing the connexin expression. This is promising for use of PQs in gap junction-mediated intercellular transfer of toxic effects in multiple systems and the bystander effect. The decrease in survivin distribution of PQ treated tumor cells also indicates good prognosis for patients post treatment since survivin is highly expressed in a range of human tumors and its expression directly correlates with both accelerated relapse and chemotherapy resistance [24]. Future studies will focus on potentiating other antineoplastic drugs through the enhancement of gap junctional activity, as well as expanding the treatment period to 21 or 28 days and look at the reoccurrence of tumors post treatment remission.

The growth suppressive effect of PQs has been previously established in multiple breast cancer cell lines [8], [9]. The antitumor effects of PQs on breast cancer xenografts in combination with antineoplastic agents in nude mice show exciting results, demonstrating that PQs alone can attenuate tumor growth. The increase in cisplatin efficacy by PQ is not additive due to PQs significant effect on tumor growth; interestingly cisplatin decreases cell communication to antagonize connexin expression and PQ. The combinational treatment of PQs and antineoplastic drugs show promising treatment for breast cancer, showing that the efficacy of antineoplastic compounds can be increased via enhancement of gap junctions. The outcome of our findings has introduced a new class of anticancer drugs, enhancing current treatment for breast cancer.
